# Real-time monitoring to predict depressive symptoms: study protocol

**DOI:** 10.3389/fpsyt.2024.1465933

**Published:** 2025-03-05

**Authors:** Yu-Rim Lee, Jong-Sun Lee

**Affiliations:** Department of Psychology, Kangwon National University, Chuncheon-si, Republic of Korea

**Keywords:** digital phenotyping, depressive disorder, ecological momentary assessment, wearable device, multilevel modeling, machine learning

## Abstract

**Introduction:**

According to the World Health Organization, Depression is the fourth leading cause of global disease burden. However, traditional clinical and self-report assessments of depression have limitations in providing timely diagnosis and intervention. Recently, digital phenotyping studies have found the possibility of overcoming these limitations through the use of wearable-devices and smartphones. The present study aims to identify the digital phenotype that significantly predicts depressive symptoms.

**Methods and analysis:**

The study will recruit a total of 150 participants in their 20s who have experienced depression for the past two weeks in Korea. The study will collect passive (eg., active energy, exercise minutes, heart rate, heart rate variability, resting energy, resting heart rate, sleep patterns, steps, walking pace) data and Ecological Momentary Assessment (EMA) through smartphone and wearable-device for two weeks. This study will be conducted longitudinally, with two repeated measurements over three months. Passive data will be collected through sensors on the wearable-device, while EMA data will be collected four times a day through a smartphone app. A machine learning algorithm and multilevel model will be used to construct a predictive model for depressive symptoms using the collected data.

**Discussion:**

This study explores the potential of wearable devices and smartphones to improve the understanding and treatment of depression in young adults. By collecting continuous, real-time data on physiological and behavioral patterns, the research uncovers subtle changes in heart rate, activity levels and sleep that correlate with depressive symptoms, providing a deeper understanding of the disorder. The findings provide a foundation for further research and contribute to the advancement of digital mental health. Advances in these areas of research may have implications for the detection and prevention of early warning signs of depression through the use of digital markers.

## 1 Introduction

Depression is associated with changes in behavioral and cognitive factors, including alterations in sleep patterns, appetite, decreased activity due to low energy, and manifestations of agitation and procrastination resulting from psychomotor changes (1). As reported by the World Health Organization (WHO) in 2023, the estimated number of individuals affected by depression is approximately 280million, equivalent to 3.8% of the world’s population (2). Alarmingly, in 2020 the prevalence of depression in South Korea is 36.8%, the highest among OECD countries. The National Health Insurance Service (NHIS) has also reported a steady increase in the number of individuals experiencing depression since 2018, surpassed 1 million cases in 2022 (3). Specifically, the percentage of young adults in their 20s and 30s is notably high, reaching 36%, according to the Health Insurance Review & Assessment (HIRA) (4). Depression is resulting in significant disability in people’s daily life and at both diagnosable and subthreshold levels, causes very high society and economic cost (5, 6). Not only is depression recognized as a significant predictor of suicidal behavior, but it is also linked to a high mortality rate among individuals diagnosed with major depressive disorder (1, 7). Consequently, depression is a highly perilous psychological disorder strongly correlated with both suicide attempts and completed suicides (8). Also, Given that depression is characterized by a chronic or recurrent course and a very high rate of relapse (9), it is crucial to promptly diagnose depression and implement early interventions to mitigate the associated risks.

Traditional assessments for diagnosing depression have relied on clinician-conducted interviews and self-report questionnaires. Nevertheless, it has limitations that render timely diagnosis and intervention challenging, as well as being time-consuming, costly, and susceptible to recall bias (10, 11). Consequently, existing assessment tools are not well-suited for detecting psychological factors that fluctuate from moment to moment. Additionally, the majority of patients seek assistance from clinicians only after their symptoms have progressed to a certain level of severity and become noticeable (12). As a result, despite the availability of a broad spectrum of empirically supported treatments for depression, the average percentage of patients achieving complete remission with no significant residual symptoms after psychotherapy is only approximately 46% (13). Hence, the need for methods to continuously monitor symptoms of depression for early diagnosis, intervention, and prevention has been highlighted (14).

In this context, there has recently been a growing body of study on digital phenotyping using mobile sensors as an adjunct tool to traditional methods for diagnosing and managing depression (9–11). Digital phenotyping, as defined by Torous, Kiang (15), involves the continuous measurement and quantification of an individual’s personal phenotype in real-time using data collected from smartphones and other personal digital devices. Smartphones and wearable devices can passively and continuously collect information such as GPS location, heart rate, step count, light levels, and more. Moreover, smartphone apps offer a more ecological approach to gather Ecological Momentary Assessment, involving the immediate collection of symptoms and behaviors reported by individuals in their natural environments (16). That is, digital phenotyping data can be utilized to monitor an individual’s depression symptoms in real-time, capturing changes from moment to moment and enabling timely intervention and diagnosis (17).

In fact, there is a growing body of evidence correlating cognitive, psychological, and physiological symptoms of depression with digital variables collected by smartphones and wearables, such as GPS data, heart rate variability, sleep patterns, and ecological momentary assessments. For instance, Saeb, Zhang (18) found a significant correlation between smartphone usage characteristics and Patient Health Questionnaire-9 (PHQ-9) scores, noting that individuals with higher depression scores tended to spend more time at home and visit fewer place. In addition, the link between depression and heart rate variability (HRV) is beginning to be explored, based on its association with abnormalities in the hypothalamic-pituitary-adrenal axis, one of the biological causes of depression, and the associated dysregulation of the autonomic nervous system (19). A study by Agelink, Boz (20) reported that patients with major depression had significantly lower HRV than the control group. Similarly, Jo, Lee (21) found a significant negative correlation between HRV and PHQ-9 scores in a healthy general population (21). In addition, Li, Liu (22) investigated the relationship between R-R intervals (RRI), measured by photoplethysmography (PPG) sensors in smartwatches, and depressive episodes. Over a period of 35 days, the research team continuously tracked participants’ RRI data and later developed a predictive model to classify depressive episodes based on post-study scores on the PHQ-9. The model achieved an average accuracy of 72%, with a maximum accuracy of 88%. These results highlight the potential of RRI data from wearable devices to capture daily fluctuations in emotional arousal and state and suggest a significant link between micro-level emotional experiences and macro-level diagnosis of depressive episodes. These findings highlight the utility of physiological markers measured by wearable devices in exploring the relationship between physiological symptoms of depression and digital phenotyping data, and provide a basis for further research.

Changes in sleep patterns, a known risk factor for depression, can also be measured by wearable devices. Several studies have examined the relationship between depressive symptoms and sleep pattern variables (23, 24). By analyzing sleep pattern characteristics collected by wearable devices and their association with depression scores, these studies found that changes in sleep architecture, sleep instability, poor sleep quality, insomnia, and increased hypersomnia were predictive of depression scores (23, 24). Further, Lim, Yun (25) examined the relationship between sleep variables measured by wearable devices and depression scores in a sample of undergraduate and graduate students. The results showed that greater intraindividual variability in sleep efficiency was associated with higher levels of depression, and a nonsignificant association was found between objectively measured sleep efficiency and subjectively measured sleep quality (25). These findings suggest that sleep variables measured by wearable devices are effective in predicting an individual’s mental health, and may be more efficient than subjective measures (e.g. Pittsburgh Sleep Quality Index: PSQI) in detecting depression in particular.

De la Barrera, Arrigoni (26) asked participants in early adulthood to respond to EMA prompts six times a day for seven days, and used the data to build a model to predict participants’ levels of depressive symptoms. The EMA prompts included questions about levels of positive and negative emotions and emotion regulation strategies. The results showed that negative emotions and the emotion regulation strategies of rumination, suppression, and distraction predicted worsening of depressive symptoms, while positive emotions and the emotion regulation strategies of problem solving, positive reappraisal, acceptance, and social sharing predicted remission of depressive symptoms (26). This suggests that using the EMA to measure and monitor behavior and mood in a natural setting may be a useful way to predict changes in depressive symptom severity in advance and provide targeted interventions. Furthermore, recent studies have combined wearable and smartphone sensor data with participant self-reports from ecological momentary assessments. In this regard, Moshe, Terhorst (12) used machine learning analytics methods to identify predictive models for depression scores by measuring smartphone sensor data (battery, GPS, screen usage, and time of day), wearable device sensor data (activity, sleep, and HRV), and ecological momentary assessment data (valence: positive/negative, arousal: low/high). The study found that the combination of smartphone, wearable device, and ecological momentary assessment data was the most predictive. These findings are consistent with the theoretical concept that depression involves cognitive, emotional and biological symptoms, and suggest the need for research using multiple data from digital devices to build a model that comprehensively predicts depression symptoms. The significant association of digital markers with depressive symptoms that has been found in many previous studies suggests the usefulness of data collected by digital devices for the prediction of depressive symptoms. It also suggests that follow-up studies with diverse populations are needed to develop rapid and accurate techniques for predicting depression symptoms using digital phenotyping data. Therefore, this study aims to identify digital predictors of depression in Korean adults and to explore the complex interrelationships among these predictors.

## 2 Methods and analysis

### 2.1 Study design

The present study employs a longitudinal design, consisting of two repeated 14-day sessions conducted every three months (**Table 1**). Passive data will be collected unobtrusively using sensors embedded in digital devices, including active energy, exercise minutes, heart rate, heart rate variability, resting energy, resting heart rate, sleep patterns, steps, walking pace. Active data will be collected through a dedicated smartphone application. This active data will include self-report measures as well as Ecological Momentary Assessment (EMA) techniques, allowing for real-time assessment of the participants’ current emotional and cognitive states in their natural environments. This protocol was approved by the Institutional Review Board of Kangwon National University (KWNUIRB-2023-02-008-001).

**Table 1:** Schedule for passive and active assessment.

	Baseline	Study Period 14 days				Endpoint
		Moring	Noontime	Evening	Night	
Active assessment						
Self-report	O					O
EMA		O	O	O	O	
Passive assessment						
Apple watch(active energy, exercise minutes, heart rate, heart rate variability, resting energy, resting heart rate, sleep patterns, steps, walking pace)		O	O	O	O	

### 2.2 Participants

We will recruit 150 individuals in their 20s who are experiencing depressive symptoms, via posts on online communities and social media sites. To be included in the study, participants must meet the following criteria: (a) score 5 or higher on the depression screening tool, PHQ-9; (b) meet diagnostic criteria for a Major Depressive Disorder (MDD) using the Structured Clinical Interview for DSM-5 Disorders Clinician Version (SCID-5-CV); (c) be at least 19 years of age; and (d) own an iOS smartphone. Exclusion criteria are under 19 years of age or over 30 years of age; have not agreed to participate in the study; questionnaire responses are random or incomplete; have suicidal ideation and plans requiring immediate attention; have current psychotic or manic states and substance use disorders; or are currently receiving other psychotherapy or counseling.

### 2.3 Procedure

#### 2.3.1 Recruitment

**Figure 1:**
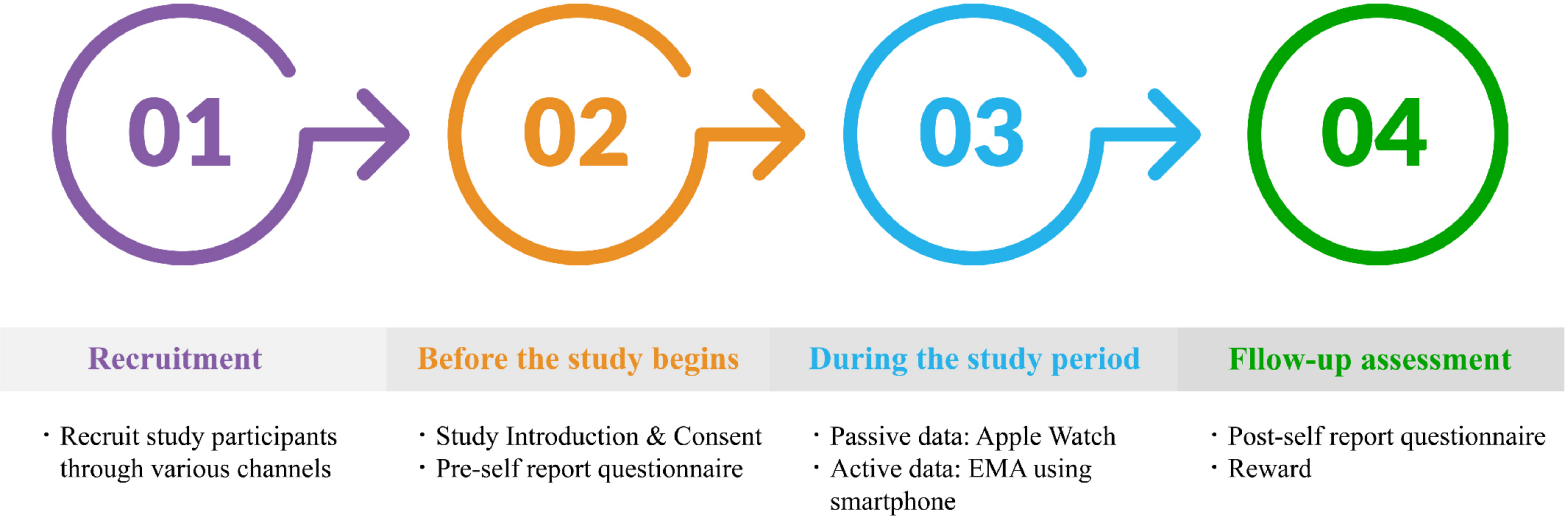
Study procedure.

We will recruit study participants through various social media platforms, such as Instagram and university online message boards (**Figure 1**). Individuals interested in the study will be able to respond to an online screening questionnaire via a QR code embedded within the study recruitment poster. The screening questionnaire collects demographic information, including age, gender, highest level of education, current economic status (upper, upper-middle, middle, lower-middle, lower), family structure (living with parents, living with one parent, living with spouse or partner, living alone), past medication history, and comorbidity, along with the PHQ-9 questionnaire. Those with a PHQ-9 score of 5 or higher on the screening questionnaire will undergo the SCID-5 to identify final participants who meet the diagnostic criteria for MDD.

#### 2.3.2 Informed consent

Participants who meet the inclusion criteria will receive a detailed explanation of the study process and informed consent. Research participants will be fully informed of the aims of the research and will be given the opportunity to voluntarily agree or refuse to participate. Participants will be able to withdraw from the study at any time. In addition, before consent is obtained, we will explain to participants that their participation in the study is not expected to result in any risks or disadvantages. However, should any physical or mental health problems arise during the completion of the questionnaires or after the experiment, the research team will refer participants to the nearest psychiatric unit of a general hospital or local counselling center for assessment and treatment. In addition, all data will be handled securely to ensure privacy. All data collected will be anonymized, with personal identifiers removed prior to analysis. Instructions for study participation will be delivered online via video. The manual will provide guidance on using the application for responding to an EMA and wearing the wearable device.

#### 2.3.3 Study duration

Participants will complete a pre-questionnaire via the mobile app developed for this study the day before the commencement of the 14-day EMA collection period. Once the study begins, participants will receive four daily reminders (morning, noon, evening, and night) from the mobile app developed for this study to complete the EMA questionnaire. Simultaneously, for passive data collection, participants will wear a wearable watch throughout the day.

#### 2.3.4 End of study

On the final day of the study, participants will again answer the same post-questionnaire as the pre-questionnaire. Participants will be compensated according to the criteria outlined in the study. Additionally, they will be informed that the same study process will be repeated in three months.

### 2.4 Measures

#### 2.4.1 Passive data

Passive data collection is performed on the Apple Watch, capturing key physiological metrics such as active energy expenditure, exercise duration, heart rate, heart rate variability (HRV), resting energy, resting heart rate, sleep patterns, step count, and walking pace. Apple Watch is equipped with advanced sensors, including a 3-axis accelerometer, gyroscope, magnetometer, optical PPG-based heart rate sensor, altimeter, ambient light sensor, temperature sensor, ECG, and capacitive touch sensors (27). These sensors provide reliable and valid measurement of multiple physiological digital markers. Comparative studies of wearable devices have shown that Apple Watch has the lowest error rate in measuring heart rate and energy expenditure (28). In addition, HRV estimates from the Apple Watch show high reliability and agreement (coefficients > 0.9) when compared to chest strap heart rate monitors (29). These findings suggest that physiological and physical data collected by the Apple Watch have significant potential for application in clinical settings.

We will encourage participants to wear the Apple Watch at all times except when showering and charging. This study will only approve and use data that Apple has officially given us access to and will ensure that we do not violate the rights and privacy of individuals. In addition, participants’ identifying information will be anonymized in the form of a participant number and uploaded via a secure process in accordance with the law.

#### 2.4.2 Active data

Both self-report assessments and Ecological Momentary Assessments (EMA) will be conducted through the will be conducted through the mobile app, currently under development by the research team. The app will provide users with mood and sleep reports based on their responses to self-report assessments and Ecological Momentary Assessments (EMA). These reports will allow users to monitor their emotional state and gain insight into the social, psychological, and physiological factors that may influence psychological distress. In addition, the app will include theoretical concepts related to depression and information on treatment methods. It will also provide contact details, such as phone numbers and website links, for national agencies where users can quickly seek help in a mental health crisis.

##### 2.4.2.1 Self-report assessment

Self-report assessments will be measured twice at the baseline and endpoint of the study and will consist of the following eight items. The baseline assessment is critical for identifying individual differences within the sample. Participants may begin the study with varying levels of depression, anxiety, or resilience. This baseline data allows us to account for these differences in our analysis. By accounting for such variability, the study can provide a more accurate understanding of how different psychological states may influence outcomes. Furthermore, comparing baseline data enables us to quantitatively track individual changes over the course of the study by comparing it with endpoint measures. This comparison allows us to explore the psychological and physiological variables that may have influenced these changes, providing insight into the factors that contribute to mental and physical health outcomes. By incorporating both initial differences and longitudinal changes, we can develop a more comprehensive understanding of the factors influencing participants outcomes throughout the study period.

The Patient Health Questionnaire-9 item (PHQ-9) (30) will be used to measure depression symptoms. The Visual Analog Scale (VAS) will be used to measure participants’ overall mood. The Functional Social Support Questionnaire (FSSQ) (31) will be used to measure participants’ perceptions of their social interactions and relationships. The Brief Resilience Scale (BRS) (32) will be used to measure participants’ resilience levels. The Generalized Anxiety Disorder 7-item scale (GAD-7) (33) will be used to measure anxiety symptoms. The WHO-Five Well Being Index will be used to measure participants’ overall happiness. The Pittsburgh Sleep Quality Index (PSQI) (34) will be used to measure participants’ sleep quality. The Primary Care-PTSD-5 (PC-PTSD-5) (35) will be used to measure symptoms of post-traumatic stress disorder. The Automatic Thoughts Questionnaire (ATQ-N) (36) will be used to measure participants’ negative thoughts in daily life. The Positive Automatic Thoughts Questionnaire (ATQ-P) (37) will be used to measure participants’ positive thoughts in daily life. The Rosenberg Self-Esteem Scale (RSE) (38) will be used to measure participants’ self-esteem. The UCLA Loneliness Scale Version 3 (39) will be used to measure loneliness as a chronic trait in participants. The Responses to Positive Affect (RPA) (40) will be used to measure how participants respond to positive emotions. The Response Style Questionnaire (RSQ) (41) will be used to measure how participants respond to depressive emotions.

##### 2.4.2.2 EMA

EMA involves four daily assessments over a 14-day period via the mobile app (**Table 2**). We utilize Ecological Momentary Assessment (EMA) data to enable real-time data collection, reducing recall bias and capturing immediate emotional responses for a more accurate depiction of daily mood fluctuations. The high frequency of data points provides detailed insights into short-term and long-term symptom patterns, allowing for a better understanding of depressive symptom dynamics (42). Additionally, EMA offers personalized insights by identifying individual differences in how participants experience and respond to daily stressors, contributing to a nuanced understanding of depression in daily life.

**Table 2:** EMA set.

	Moring	Noontime	Evening	Night
Previous night’s sleep status	O			
Mood & RPA		O	O	O
Daily diary				O

Sleep Status Questionnaires: In addition to collecting objective sleep data through wearable devices, we will also gather subjective self-reported sleep data from participants. This dual approach will allow us to correlate subjective and objective sleep data, providing a comprehensive understanding of each participant’s sleep experience. Sleep status will be measured once a day, every morning. Participants will complete a brief survey on their smartphones, which will include questions about the previous night’s sleep. These questions will cover the time they went to bed, the duration it took to fall asleep, the time they woke up, the total amount of sleep they obtained, and their subjective sleep quality (‘1=very poor’ to ‘4 =very good’). By combining the objective data from the wearables with the subjective self-reports, we aim to gain insights into the consistency between perceived and actual sleep patterns and understand the factors influencing subjective sleep quality.

Mood Questionnaires: Participants will receive prompts to respond to mood-related questions three times a day—at lunch (12:00~17:59), dinner (18:00~20:59), and night (21:00~23:00). Using a Visual Analog Scale (VAS) ranging from 0 (not at all) to 8 (very much), participants will rate their current mood on 10 negative emotions (depression, anxiety, sadness, fear, loneliness, rejection, anger at self, anger at others, emptiness, shame) and 4 positive emotions (joy, pleasure, satisfaction, relaxation). The Visual Analog Scale (VAS) has been widely recognized as a reliable, valid, and user-friendly instrument for assessing subjective states, including mood (43). Furthermore, studies have demonstrated a significant correlation between depression scores measured by the VAS and both the total and individual item scores on the PHQ-9, reinforcing its applicability in clinical and research settings (44).This detailed approach allows us to gather nuanced data on the intensity and frequency of various moods.

Responses to Positive Affect Questionnaires: In this study, participants will respond to the Responses to Positive Affect Questionnaires(RPA) (40) via a smartphone app three times a day - at lunch, dinner, and night. The survey will ask them to reflect on how they have responded to positive emotions since their last response. Specifically, participants will report whether they have primarily engaged in blocking out positive moods or focusing on positive moods. This method allows for the collection of real-time data on emotional responses, providing a nuanced understanding of how individuals process positive experiences throughout the day. In addition, by analyzing the data collected, the study will determine whether certain patterns of managing positive emotions are associated with higher or lower levels of depressive symptoms.

Depressive Symptoms Questionnaires: In this study, participants will complete the Clinically Useful Depression Outcome Scale (CUDOS-D) (45) once each night to report depressive symptoms experienced during the day. The CUDOS-D consists of 18 items assessing diagnostic criteria for major depressive disorder (MDD), psychosocial impairment, and quality of life. Each item is rated on a 5-point Likert scale (0 = not at all, 1 = a little, 2 = moderately, 3 = quite a bit, 4 = extremely). Specific symptoms assessed by the CUDOS-D include: depressed mood, loss of interest in usual activities, low energy, psychomotor agitation, psychomotor retardation, guilt, worthlessness, thoughts of death, suicidal ideation, difficulty concentrating, indecisiveness, decreased appetite, increased appetite, insomnia, hypersomnia, and hopelessness. By collecting data on a daily basis, the CUDOS-D allows for detailed tracking of symptom fluctuations over time, providing valuable insight into participants’ mental health trajectories.

Daily Diary: Participants will receive a reminder each night to reflect on their day’s events and their emotional responses. The diary entry will begin with participants freely writing about their overall day, focusing on activities and interactions that shaped their experiences. Following this general reflection, participants will be prompted to identify a specific event that had a significant impact on their emotions. They will detail this event, describing why it stood out and how it influenced their mood. The smartphone app will streamline data collection by ensuring consistent and timely entries, while also facilitating a user-friendly platform for participants to express their thoughts and feelings. This methodology aims to capture real-time emotional processes and coping mechanisms, providing rich, qualitative data on how daily events shape psychological well-being.

### 2.5 Analyses

To analyze the Apple Watch passive data and longitudinal data from the Ecological Momentary Assessment (EMA), we will employ a comprehensive, multi-pronged approach. This includes multi-model analyses, along with basic data analysis methods.

#### 2.5.1 Basic analyses

To analyze this longitudinal data, we will employ a range of basic statistical techniques, including t-tests, repeated measures ANOVA, and linear regression analysis. These methods will enable us to identify significant differences within the data and explore within-subject variations over time. Additionally, we will examine associations between the passive data collected from the Apple Watch and the self-reported EMA data to identify potential predictors and better understand the interaction between physical and mental variable.

To mitigate the influence of confounding variable on digital phenotype, we will additionally conduct multivariate analyses (e.g., regression analysis) that include not only demographic variables but also medication use and comorbidity status when examining their associations with digital phenotypes. If necessary, we can conduct subgroup analyses to compare digital phenotypes across groups with and without medication use or specific comorbidities. This will help us to clarify the influence of confounding variables. We will endeavor to control the influence of confounding variables and analyze the net association between digital phenotypes and other variables of interest using statistical techniques such as propensity score matching, where appropriate.

#### 2.5.2 Multi model analyses

Dynamic Structural Equation Modeling (DSEM) is an advanced statistical technique that integrates time-series analysis with structural equation modeling (46). In this study, we employ a two-level DSEM approach, specifically utilizing a multilevel Vector Autoregressive (VAR) model to explore the temporal relationships between various predictors and depressive mood scores. This method is particularly effective for handling nested data structures, such as repeated measures within individuals over time. DSEM allows for the separation of within-individual (Level 1) and between-individual (Level 2) variability, which enables us to examine how a predictor like step count (at time *t*-1) affects depressive mood scores at a later time point (e.g., time *t*) at both levels.

At the first level (within-individual), we apply a multilevel VAR model to capture the temporal dependencies between depressive mood and several physiological and behavioral predictor variables. This model is highly useful for understanding the causal relationships and interactions between variables measured repeatedly over time. Specifically, predictors such as step count, heart rate, heart rate variability, and sleep quality are hypothesized to be strongly associated with fluctuations in depressive mood over time. For instance, we evaluate how the total number of steps taken by a participant on one day (*t*-1) influences their depressive mood the next day (*t*). Similarly, physiological measures like heart rate and heart rate variability (47), which reflect stress or emotional states, are examined to assess their immediate or delayed impact on depressive mood. Sleep quality is also a key factor; poor sleep quality on one night (*t*-1) can lead to heightened depressive symptoms the following day (*t*). Bayesian estimation techniques are employed to model these relationships, providing robust estimates of the temporal correlations and accounting for the uncertainty between variables. This method increases the reliability of the analysis, allowing us to capture individual changes over time. These insights are crucial for developing personalized intervention strategies.

At the second level (between-individual), we model how these temporal relationships vary across participants. This approach accounts for the fact that the relationship between a predictor (e.g., step count) and an outcome (e.g., depressive mood) may differ between individuals. For example, while one participant may experience a strong association between step count and mood, another participant might show little to no effect. To capture these individual differences, a random coefficients model is used, allowing us to model variability in the estimated coefficients across participants. This analysis highlights how individual factors such as depression severity, gender, age, and lifestyle habits might influence the strength of these relationships. The two-level analysis provides valuable insights into interindividual differences, offering a deeper understanding of the heterogeneity in the temporal dynamics of depressive symptoms. The results can inform tailored intervention strategies that account for individual differences. For instance, for a participant who shows a strong relationship between step count and depressive mood, increasing physical activity may be an effective intervention. Conversely, for someone whose depressive mood is more closely linked to heart rate variability, a stress management program may be more appropriate. In conclusion, this two-level analysis approach not only uncovers important individual differences but also enables the design of more personalized and sophisticated mental health interventions.

## 3 Discussion

This study has several important implications for using smartphones and wearables to understand and intervene in depression among 20-somethings. First, it improves our understanding of the complex relationship between physiological and behavioral patterns and depressive symptoms. This will allow healthcare providers to identify physiological and behavioral patterns associated with depression and integrate personalized monitoring systems into treatment plans for more precise and immediate interventions. This continuous monitoring will not only help track symptoms, but will also provide real-time feedback to both patients and healthcare providers, helping them to tailor treatment strategies for more timely interventions based on the patient’s current state. In addition, subtle changes in patterns such as heart rate, sleep disturbances and activity levels can be detected through continuous real-time data collection, providing an opportunity for proactive mental health intervention. The findings suggest that by identifying early warning signs of a depressive episode, clinicians may be able to intervene at an early stage, potentially preventing worsening of symptoms. The results of this study will serve as a basis for further research and contribute to the advancement of the field of digital mental health. Also, the potential for personalized mental health interventions has been greatly enhanced. Personalized interventions based on real-time data could be integrated into digital health platforms to provide users with immediate coping strategies or therapeutic exercises when symptoms are detected. This approach could reduce the burden on existing healthcare systems by providing a scalable and cost-effective solution for ongoing mental health care.

In addition, smartphone-based Ecological Momentary Assessment (EMA) utilized in this study presents distinct advantages in capturing psychosocial data while minimizing disruption to participants’ natural environments. Unlike traditional assessment methods, EMA facilitates continuous, real-time monitoring of participants’ emotional and behavioral changes, offering data that are ecologically valid. By leveraging smartphones, which are ubiquitous in daily life, we are able to automatically collect data without imposing additional burdens on participants, thus enhancing the feasibility and compliance of long-term studies (16, 48). One key advantage of EMA is its ability to reduce recall bias. Traditional self-report methods often suffer from participants’ memory distortions over time. EMA, on the other hand, prompts participants to respond in the moment, thereby collecting data that more accurately reflects their immediate experiences (48). This feature is particularly valuable for mental health research, where real-time monitoring of affective states and behaviors can provide critical insights into fluctuations in mood and behavioral patterns (49). Furthermore, EMA fosters self-awareness among participants by encouraging them to regularly track their mental and emotional states. This increased self-monitoring can have direct therapeutic benefits, promoting greater engagement in their own mental health management. Given these strengths, smartphone-based EMA stands out as a highly effective tool not only for research but also for clinical interventions aimed at monitoring and managing mental health disorders.

In conclusion, this study underscores the transformative potential of advanced technology in revolutionizing mental health care. By leveraging wearables and smartphones, researchers can gain unprecedented insights into the psychophysiological underpinnings of depression, leading to more effective and personalized interventions. However, integration with clinical practice is a critical area for further discussion (50). It is crucial to explore how these findings can be incorporated into existing clinical workflows. Researchers must understand how to use this data to complement traditional diagnostic methods and therapeutic interventions, ensuring that the benefits of continuous monitoring are fully realized in practice. Subsequent studies can build upon these initial findings, refining and expanding the knowledge base in this emerging field, ultimately paving the way for more effective and personalized interventions for individuals with depression.

While this study provides valuable insights into digital phenotyping for predicting depression among young adults, several limitations should be noted. Firstly, the sample size of 150 participants, while sufficient for initial exploration, may limit the generalizability of our findings. Future studies should aim to recruit larger, more diverse samples to enhance statistical power and representativeness. Secondly, our focus on young adults in their 20s may not capture the full spectrum of depression across different age groups. Expanding the age range in future research could provide a more comprehensive understanding of digital phenotyping across the lifespan. Thirdly, while we collected a range of demographic data, including age, gender, education level, economic status, and family structure, there may be other relevant factors not captured in our study. Future research could explore additional demographic variables or environmental factors that might influence digital phenotyping patterns. Fourthly, while our longitudinal design with three time points (T1, T2, T3) provides valuable insights into changes over time, a longer follow-up period or more frequent assessments could offer a more correct understanding of the temporal dynamics of digital phenotyping in relation to depression. Lastly, our study was conducted in a specific cultural and geographical context, which may limit the generalizability of our findings to other populations or cultures. Cross-cultural studies or multi-site research could help determine the universality of digital phenotyping patterns for depression prediction.

Despite these limitations, the findings of our study have significant potential to inform and enhance clinical practice in mental health, particularly in the development of digital mental health interventions. Firstly, the digital phenotyping patterns identified in our research could be used to develop more personalized and timely interventions for young adults at risk of depression. For instance, smartphone applications could be designed to detect early warning signs based on changes in digital behavior, prompting targeted interventions or suggesting professional help when needed. Secondly, our results could inform the creation of AI-driven chatbots or virtual assistants that can provide real-time support and resources to individuals showing signs of depressive symptoms. These digital tools could offer coping strategies, mindfulness exercises, or connections to mental health professionals based on the user’s digital phenotype. Thirdly, the longitudinal nature of our data collection (T1, T2, T3) provides insights into the progression of depressive symptoms over time. This information could be valuable in developing predictive models for relapse prevention, allowing clinicians to intervene proactively before symptoms worsen. Furthermore, our findings on the relationship between physical activity, sleep patterns, and depressive symptoms could be integrated into existing health tracking applications. These apps could then provide more holistic health recommendations that consider both physical and mental well-being. Lastly, the digital phenotyping approach could be extended to other mental health conditions, potentially revolutionizing how we diagnose and treat a range of psychological disorders. This could lead to more efficient, accessible, and cost-effective mental health care delivery, especially in areas with limited access to traditional mental health services. While these applications hold great promise, it’s crucial to emphasize the need for rigorous testing and validation of any digital mental health interventions before widespread implementation. Future research should focus on translating these findings into practical, ethical, and effective digital tools that can complement existing mental health care practices.

## References

[1] APA . Diagnostic and Statistical Manual of Mental Disorders: Dsm-5. Washington, DC: American psychiatric association (2013).

[2] WHO . Depressive disorder (2023). Available online at: https://www.who.int/news-room/fact-sheets/detail/depression (Accessed March 31, 2023).

[3] NHIS . National Health Insurance Service_Depression Bipolar Disorder Schizophrenia Practice Statistics_20230630. Wonju, Gangwon Special Self-Governing Province, Republic of Korea: National Health Insurance Service (2023).

[4] HIRA . 2023_Disease and Medical Behaviors in Everyday Life Statistics. Wonju, Gangwon Special Self-Governing Province, Republic of Korea: Health Insurance Review & Assessment Service (2023).

[5] Lewinsohn PM Solomon A Seeley JR Zeiss A . Clinical implications of” Subthreshold” Depressive symptoms. J Abnormal Psychol. (2000) 109:345. doi: 10.1037/0021-843X.109.2.345 10895574

[6] Martin J Blum T Beach S Roman P . Subclinical depression and performance at work. Soc Psychiatry Psychiatr Epidemiol. (1996) 31:3–9. doi: 10.1007/BF00789116 8821918

[7] Rihmer Z . Suicide risk in mood disorders. Curr Opin Psychiatry. (2007) 20:17–22. doi: 10.1097/YCO.0b013e3280106868 17143077

[8] Mościcki EK . Epidemiology of completed and attempted suicide: toward a framework for prevention. Clin Neurosci Res. (2001) 1:310–23. doi: 10.1016/S1566-2772(01)00032-9

[9] Onnela JP Rauch SL . Harnessing smartphone-based digital phenotyping to enhance behavioral and mental health. Neuropsychopharmacology. (2016) 41:1691–6. doi: 10.1038/npp.2016.7 PMC4869063 26818126

[10] Insel TR . Digital phenotyping: technology for a new science of behavior. JAMA. (2017) 318:1215–6. doi: 10.1001/jama.2017.11295 28973224

[11] Opoku Asare K Moshe I Terhorst Y Vega J Hosio S Baumeister H . Mood ratings and digital biomarkers from smartphone and wearable data differentiates and predicts depression status: A longitudinal data analysis. Pervasive Mobile Computing. (2022) 83:1–13. doi: 10.1016/j.pmcj.2022.101621

[12] Moshe I Terhorst Y Opoku Asare K Sander LB Ferreira D Baumeister H . Predicting symptoms of depression and anxiety using smartphone and wearable data. Front Psychiatry. (2021) 12:625247. doi: 10.3389/fpsyt.2021.625247 33584388 PMC7876288

[13] Casacalenda N Perry JC Looper K . Remission in major depressive disorder: A comparison of pharmacotherapy, psychotherapy, and control conditions. Am J Psychiatry. (2002) 159:1354–60. doi: 10.1176/appi.ajp.159.8.1354 12153828

[14] Zarate D Stavropoulos V Ball M de Sena Collier G Jacobson NC . Exploring the digital footprint of depression: A prisma systematic literature review of the empirical evidence. BMC Psychiatry. (2022) 22:421. doi: 10.1186/s12888-022-04013-y 35733121 PMC9214685

[15] Torous J Kiang MV Lorme J Onnela J-P . New tools for new research in psychiatry: A scalable and customizable platform to empower data driven smartphone research. JMIR Ment Health. (2016) 3:e5165. doi: 10.2196/mental.5165 PMC4873624 27150677

[16] Shiffman S Stone AA Hufford MR . Ecological momentary assessment. Annu Rev Clin Psychol. (2008) 4:1–32. doi: 10.1146/annurev.clinpsy.3.022806.091415 18509902

[17] Sharma CM Chariar VM . Diagnosis of mental disorders using machine learning: literature review and bibliometric mapping from 2012 to 2023. Heliyon. (2024) 10(12):1–23. doi: 10.1016/j.heliyon.2024.e32548 PMC11225745 38975193

[18] Saeb S Zhang M Karr CJ Schueller SM Corden ME Kording KP . Mobile phone sensor correlates of depressive symptom severity in daily-life behavior: an exploratory study. J Med Internet Res. (2015) 17:e175. doi: 10.2196/jmir.4273 26180009 PMC4526997

[19] Jung C-H M J-A Seo H-J Chae J-H . The relationship between heart rate variability and symptom severity in patients with major depressive disorder. Mood Emotion. (2010) 8:120–5.

[20] Agelink MW Boz C Ullrich H Andrich J . Relationship between major depression and heart rate variability.: clinical consequences and implications for antidepressive treatment. Psychiatry Res. (2002) 113:139–49. doi: 10.1016/S0165-1781(02)00225-1 12467953

[21] Jo YT Lee SW Park S Lee J . Association between heart rate variability metrics from a smartwatch and self-reported depression and anxiety symptoms: A four-week longitudinal study. Front Psychiatry. (2024) 15:1371946. doi: 10.3389/fpsyt.2024.1371946 38881544 PMC11176536

[22] Li F Liu G Zou Z Yan Y Huang X Liu X . A classification framework for depressive episode using Rr intervals from smartwatch. IEEE Trans Affect Computing. (2023) 15(3):1387–99. doi: 10.1109/TAFFC.2023.3343463

[23] Rykov Y Thach T-Q Bojic I Christopoulos G Car J . Digital biomarkers for depression screening with wearable devices: cross-sectional study with machine learning modeling. JMIR mHealth uHealth. (2021) 9:e24872. doi: 10.2196/24872 34694233 PMC8576601

[24] Zhang Y Folarin AA Sun S Cummins N Bendayan R Ranjan Y . Relationship between major depression symptom severity and sleep collected using a wristband wearable device: multicenter longitudinal observational study. JMIR mHealth uHealth. (2021) 9:e24604. doi: 10.2196/24604 33843591 PMC8076992

[25] Lim J-A Yun J-Y Choi S-H Park S Suk HW Jang JH . Greater variability in daily sleep efficiency predicts depression and anxiety in young adults: estimation of depression severity using the two-week sleep quality records of wearable devices. Front Psychiatry. (2022) 13:1041747. doi: 10.3389/fpsyt.2022.1041747 36419969 PMC9676252

[26] De la Barrera U Arrigoni F Monserrat C Montoya-Castilla I Gil-Gómez J-A . Using ecological momentary assessment and machine learning techniques to predict depressive symptoms in emerging adults. Psychiatry Res. (2024) 332:115710. doi: 10.1016/j.psychres.2023.115710 38194800

[27] Lui GY Loughnane D Polley C Jayarathna T Breen PP . The apple watch for monitoring mental health–related physiological symptoms: literature review. JMIR Ment Health. (2022) 9:e37354. doi: 10.2196/37354 36069848 PMC9494213

[28] Shcherbina A Mattsson CM Waggott D Salisbury H Christle JW Hastie T . Accuracy in wrist-worn, sensor-based measurements of heart rate and energy expenditure in a diverse cohort. J personalized Med. (2017) 7:3. doi: 10.3390/jpm7020003 PMC5491979 28538708

[29] Hernando D Roca S Sancho J Alesanco ÁChecktae Bailón R . Validation of the apple watch for heart rate variability measurements during relax and mental stress in healthy subjects. Sensors. (2018) 18:2619. doi: 10.3390/s18082619 30103376 PMC6111985

[30] Spitzer RL Kroenke K Williams JB Group. PHQPCS . Validation and utility of a self-report version of prime-Md: the Phq primary care study. JAMA. (1999) 282:1737–44. doi: 10.1001/jama.282.18.1737 10568646

[31] Broadhead W Gehlbach SH De Gruy FV Kaplan BH . The Duke-Unc functional social support questionnaire: measurement of social support in family medicine patients. Med Care. (1988) 26(7):709–23. doi: 10.1097/00005650-198807000-00006 3393031

[32] Smith BW Dalen J Wiggins K Tooley E Christopher P Bernard J . The brief resilience scale: assessing the ability to bounce back. Int J Behav Med. (2008) 15:194–200. doi: 10.1080/10705500802222972 18696313

[33] Spitzer RL Kroenke K Williams JB Lowe B . A brief measure for assessing generalized anxiety disorder: the Gad-7. Arch Intern Med. (2006) 166:1092–7. doi: 10.1001/archinte.166.10.1092 16717171

[34] Buysse DJ Reynolds CF III Monk TH Berman SR Kupfer DJ . The Pittsburgh sleep quality index: A new instrument for psychiatric practice and research. Psychiatry Res. (1989) 28:193–213. doi: 10.1016/0165-1781(89)90047-4 2748771

[35] Prins A Bovin MJ Smolenski DJ Marx BP Kimerling R Jenkins-Guarnieri MA . The primary care Ptsd screen for Dsm-5 (Pc-Ptsd-5): development and evaluation within a veteran primary care sample. J Gen Intern Med. (2016) 31:1206–11. doi: 10.1007/s11606-016-3703-5 PMC5023594 27170304

[36] Hollon SD Kendall PC . Cognitive self-statements in depression: development of an automatic thoughts questionnaire. Cogn Ther Res. (1980) 4:383–95. doi: 10.1007/BF01178214

[37] Ingram RE Wisnicki KS . Assessment of positive automatic cognition. J consulting Clin Psychol. (1988) 56:898. doi: 10.1037/0022-006X.56.6.898 3204200

[38] Rosenberg M . Rosenberg self-esteem scale. J Religion Health. (1965) 61. doi: 10.1037/t01038-000

[39] Russell DW . Ucla loneliness scale (Version 3): reliability, validity, and factor structure. J Pers Assess. (1996) 66:20–40. doi: 10.1207/s15327752jpa6601_2 8576833

[40] Feldman GC Joormann J Johnson SL . Responses to positive affect: A self-report measure of rumination and dampening. Cogn Ther Res. (2008) 32:507–25. doi: 10.1007/s10608-006-9083-0 PMC2847784 20360998

[41] Nolen-Hoeksema S . Responses to depression and their effects on the duration of depressive episodes. J Abnormal Psychol. (1991) 100:569. doi: 10.1037/0021-843X.100.4.569 1757671

[42] Mengelkoch S Moriarity DP Novak AM Snyder MP Slavich GM Lev-Ari S . Using ecological momentary assessments to study how daily fluctuations in psychological states impact stress, well-being, and health. J Clin Med. (2023) 13:24. doi: 10.3390/jcm13010024 38202031 PMC10779927

[43] Folstein MF Luria R . Reliability, validity, and clinical application of the visual analogue mood scale1. psychol Med. (1973) 3:479–86. doi: 10.1017/S0033291700054283 4762224

[44] Huang Z Kohler IV Kämpfen F . A single-item visual analogue scale (Vas) measure for assessing depression among college students. Community Ment Health J. (2020) 56:355–67. doi: 10.1007/s10597-019-00469-7 31531784

[45] Zimmerman M Harris L Martin J McGonigal P . Reliability and validity of a self-report scale for daily assessments of the severity of depressive symptoms. Psychiatry Res. (2018) 270:581–6. doi: 10.1016/j.psychres.2018.10.007 30368164

[46] Asparouhov T Hamaker EL Muthén B . Dynamic structural equation models. Struct equation modeling: Multidiscip J. (2018) 25:359–88. doi: 10.1080/10705511.2017.1406803 29624092

[47] Velmovitsky PE Alencar P Leatherdale ST Cowan D Morita PP . Using apple watch Ecg data for heart rate variability monitoring and stress prediction: A pilot study. Front Digital Health. (2022) 4:1058826. doi: 10.3389/fdgth.2022.1058826 PMC9780663 36569803

[48] Burke LE Shiffman S Music E Styn MA Kriska A Smailagic A . Ecological momentary assessment in behavioral research: addressing technological and human participant challenges. J Med Internet Res. (2017) 19:e77. doi: 10.2196/jmir.7138 28298264 PMC5371716

[49] Yang YS Ryu GW Choi M . Methodological strategies for ecological momentary assessment to evaluate mood and stress in adult patients using mobile phones: systematic review. JMIR mHealth uHealth. (2019) 7:e11215. doi: 10.2196/11215 30932866 PMC6462888

[50] Porras-Segovia A Molina-Madueño RM Berrouiguet S López-Castroman J Barrigón ML Pérez-Rodríguez MS . Smartphone-based ecological momentary assessment (Ema) in psychiatric patients and student controls: A real-world feasibility study. J Affect Disord. (2020) 274:733–41. doi: 10.1016/j.jad.2020.05.067 32664009

